# Maternal platelet-to-lymphocyte ratio at delivery can predict poor neonatal outcome in preterm births

**DOI:** 10.4274/tjod.65299

**Published:** 2019-01-09

**Authors:** Dikra Waeeb Jaffar, Maha Abubakr Feissal Rabie

**Affiliations:** 1 Aden University Faculty of Medicine, Department of Gynecology and Obstetrics, Aden, Yemen; 2 Pharos University in Alexandria, Department of Medical Laboratory Technology, Alexandria, Egypt

**Keywords:** Lymphocytes, neonatal, outcome, platelets, preterm, ratio

## Abstract

**Objective:**

To determine the role of the platelet-to-lymphocyte ratio (PLR) in predicting poor neonatal outcome among preterm births (PTB).

**Materials and Methods:**

The medical records of 439 PTBs and 200 normal pregnancies were reviewed retrospectively using some personal and obstetric data, as well as complete blood count reports.

**Results:**

There were significantly poor neonatal outcomes among PTBs in regard to birth weight, APGAR score, morbidity, and mortality. There were significantly poor outcomes for preterm neonates delivered to mothers with PLR ≥80 at delivery for low APGAR score, respiratory distress syndrome, intraventricular hemorrhage and perinatal death. There was a significant negative correlation between maternal PLR at delivery and birth weight, as well as gestational age of PTB.

**Conclusion:**

Maternal PLR at delivery has a significant relationship with neonatal outcomes. It can predict preterm neonates with poor outcomes.

**PRECIS:** Maternal platelet to lymphocytes ratio at delivery can predict poor neonatal outcome in preterm births.

## Introduction

Premature birth continues to be one of the most important challenges of modern obstetrics through its high incidence and its implications on neonatal morbidity and mortality^(1)^. Preterm birth (PTB) is one of the most common obstetric problems, and preterm neonates are more likely to die than term infants. Furthermore, those who survive run a greater risk of disability than term infants^ (2)^.

Platelets and lymphocytes share regulatory mechanisms in the pathophysiology of thrombosis, inflammation, immunity, and atherosclerosis. The effect of platelets on lymphocyte function may be via direct contact or by soluble mediators^ (3)^. Platelets enhance adhesion and cell migration of lymphocytes, and affect other functional aspects of lymphocytes in a complex manner^(4)^.

The platelet-to-lymphocyte ratio (PLR) was suggested by previous studies to be a strong predictor of inflammation ^(3,5)^. It is a good indicator of platelet activation, lymphocyte function, and immune response^(3)^ In obstetrics, the PLR was previously studied as a new inflammatory marker for the diagnosis of preterm premature rupture of membranes,^(6)^ as a predictor for severity of preeclampsia,^(7,8,9)^ and Kurtoglu et al.^(10)^ reported that it may be useful in the discrimination of benign and malignant endometrial lesions, and early and advanced-stage endometrial cancer.

Klement et al.^ (11)^ conducted a population-based study on the PLR among pregnant women. They found a maximum PLR value during the second trimester, which showed a positive correlation with maternal age. However, no differences were found between the high-risk and normal population, excluding patients with a fibroid uterus or inflammatory bowel disease who presented significantly elevated PLRs through all trimesters.

This study was conducted to investigate the relationship between maternal PLR at delivery and preterm neonatal outcomes in a group of mothers selected from 2 seaside cities in 2 different countries, the first was the city of Aden located on the Arabian sea (Yemen), and the second was Alexandria city located on the Mediterranean sea (Egypt), aiming to find a benefit in using maternal PLR in predicting neonatal outcomes in PTB.

## Materials and Methods

This is a retrospective study conducted on 439 PTBs collected from Al-Sadaka Teaching Hospital in Aden (Yemen) and Al-Zohour Hospital, Sedi Beshr, in Alexandria (Egypt) during 2017. In addition, the records of 200 term pregnant women who delivered at the same hospitals during the same duration were used as a control group. Data collected included maternal age, parity, gestational age at delivery and neonatal outcome variables (birth weight, APGAR score at 5 minutes, morbidity, and mortality). The platelet count and the absolute lymphocyte count were taken from the complete blood count report at delivery, and then the PLR was calculated.

### Ethical consideration

This study was conducted retrospectively after obtaining consent from the hospitals’ directors and archive who requested to code personal data to numbers, and accordingly, there was no physical or psychological harm for the patients and controls included in this study.

### Statistical Analysis

Data were processed using the SPSS program version 24. Quantitative variables found with parametric distribution are presented as means and standard deviations with ranges. Student’s t-test was used to investigate the presence of a significant difference between the PTBs and controls. Qualitative variables were compared using the chi-square test and Fisher’s exact test, as appropriate.

A receiver operating characteristics (ROC) curve was drawn for the PLR to obtain the cutoff value with the highest sensitivity and specificity. Then Pearson rank-order correlation tests were conducted between the PLR, gestational age, and birth weight of the PTBs. All statistical tests were conducted with 95% confidence intervals and a p value of ≤0.05 was considered statistically significant.

## Results

In this study, there was no significant difference between the PTBs and controls regarding maternal age and parity. Only gestational age was statistically significantly higher among the controls. The mean lymphocyte count was significantly higher among the controls, and the mean PLR was significantly higher among the women with PTBs (Table 1).

Neonatal outcomes among the studied PTBs revealed significant poor outcome regarding birth weight, APGAR score, morbidity and mortality, when compared to the control (Table 2).

**Figure 1:**
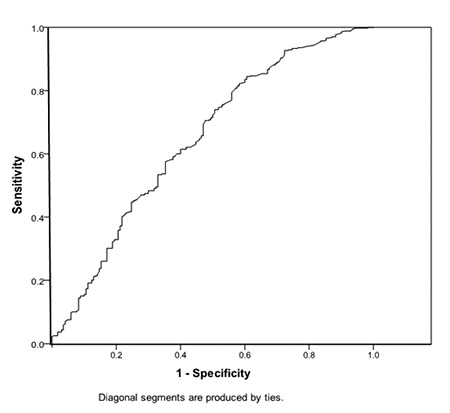
The receiver operating characteristic curve for the platelet-to-lymphocyte ratio

The ROC curve for PLR among the studied PTBs, showed significant area under the curve (AUC=0.647, p=0.026) (Figure 1). With a cutoff value of 80, there was no significant difference in the mean maternal age, parity and gestational age between the women with PTB with PLR <80 or PLR ≥80. A significant difference was evident in the mean platelet count, lymphocyte count, and PLR in both groups (Table 3).

There was significant poor outcome for preterm neonates delivered to mothers with PLR ≥80. They showed a significantly higher percentage of neonates with low APGAR score, RDS, intraventricular hemorrhage, and perinatal death (Table 4).

**Figure 2:**
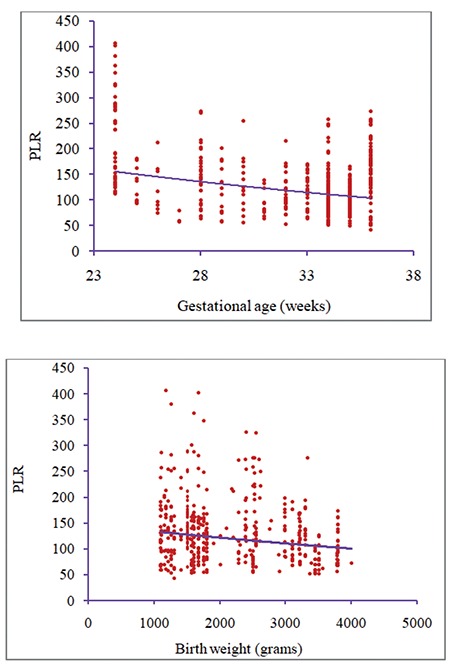
Negative correlation between the platelet-to-lymphocyte ratio and gestational age and birth weight

The Pearson rank-order correlation test showed a significant negative correlation between PLR and birth weight (r=*-*0.189, p=0.001) as well as gestational age (r=-0.345, p=0.001) among the studied PTBs (Figure 2).

## Discussion

Preterm neonates have poorly developed organ systems that put them at risk for many life-threatening conditions. They are at risk for hypothermia because they cannot produce and retain enough heat to maintain their body temperatures, respiratory distress syndrome from deficiency in surfactant production and lung development and bronchopulmonary dysplasia, cardiovascular abnormalities including patent ductus arteriosus and low blood pressure, intraventricular hemorrhage, ineffective glucose regulation, necrotizing enterocolitis, infection and retinopathy of prematurity.^(12)^

Different factors may play a role in poor obstetric outcomes such as PTB. These factors include high or low maternal age (>34 yrs and <17 yrs), smoking, alcohol or drug use during pregnancy, inadequate prenatal care, multiple pregnancies, nutritional status, co-morbidities such as hypertension, diabetes, and genitourinary tract infections, and certain biologic or genetic markers^(13,14,15)^.

In the current study, a higher perinatal mortality rate was observed among PTBs. The earlier the gestational age, the greater the risk of morbidity and death. The relationship between mortality and immaturity (i.e. early gestational age at birth) is not linear but exponential. Though only 3-4% of births occur before 34 weeks, they account for the majority of neonatal deaths^(16,17)^.

The PLR is a marker that can predict inflammation, thrombotic events, and malignancies. Previous reports showed a significant association between high PLRs and major adverse outcomes in renal diseases, and reduced survival in malignancies such as endometrial cancer^(18,19,20,21)^.

The ROC curve for PLR in this study showed a significant AUC. This significant area may help in using the PLR among PTBs to predict neonatal outcome among preterm deliveries. When the cutoff value of 80 was used, preterm neonates delivered to mothers with PRL ≥80 showed significantly poor outcomes in APGAR score, RDS, intraventricular hemorrhage, and perinatal death.

In the current study, there was significant negative correlation between maternal PLR at delivery with birth weight and gestational age of PTBs. Similar findings were reported by Akgün et al.^(22)^ in Turkey among 783 pregnant women; the authors concluded that PLR was negatively correlated with the week of birth and birth weight of the infant.

The ability to identify late-preterm infants who are prone to neonatal complications would be of great importance for counseling purposes. In the current study, maternal PLR at delivery was assessed in relation to neonatal outcomes among PTBs. Our findings suggest the use of maternal PLR at delivery as a prognostic marker for neonatal outcomes. The higher the PLR, the worse the neonatal outcomes in PTBs.

### Conclusion and recommendation

Maternal PLR at delivery has a significant relationship with neonatal outcomes. It can predict preterm neonates with poor outcomes. Further studies are recommended for PLR in early pregnancy to identify pregnant women at risk of preterm delivery, who require special prenatal follow-up and preventive therapies to reduce the number of premature births.

## Figures and Tables

**Table 1:**
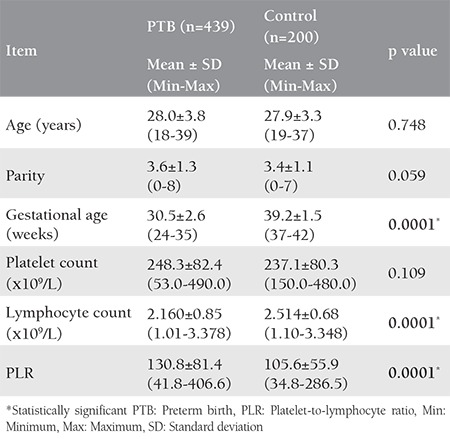
Basic data obtained from medical records of preterm births and the control

**Table 2:**
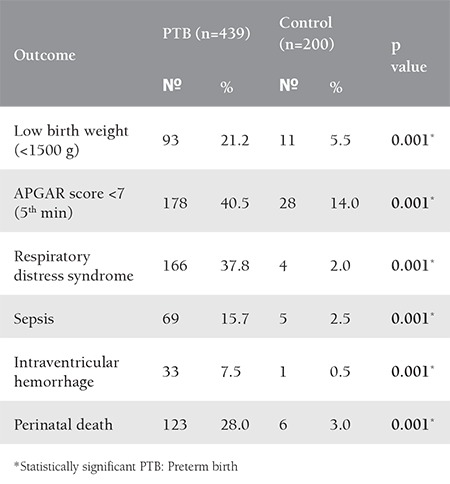
Neonatal outcomes for the preterm births and controls

**Table 3:**
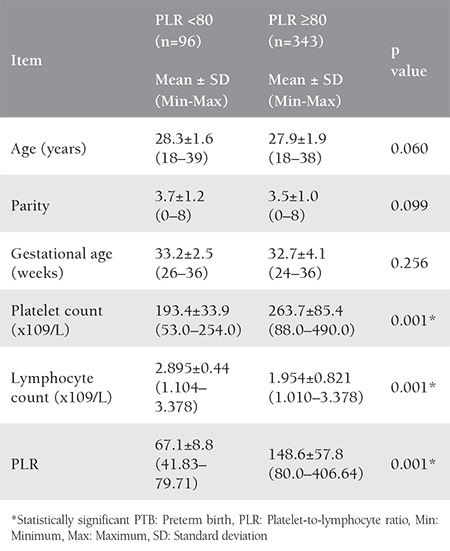
Demographic and laboratory data of pregnant women with preterm births in relation to the platelet-to-lymphocyte ratio with a cut-off value of 80

**Table 4:**
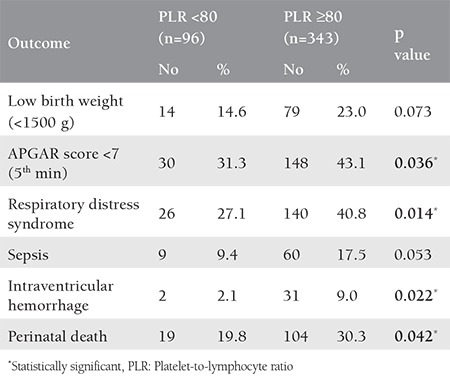
The relationship between the platelet-to-lymphocyte ratio and neonatal outcomes for preterm births with a cut-off value of 80

## References

[1] Moore ML (2002). Preterm Birth: A Continuing Challenge. The Journal of Perinatal Education.

[2] Lumley J (2003). Defining the problem: the epidemiology of preterm birth. BJOG: An Intern J Obstet Gynaecol.

[3] Li N (2008). Platelet–lymphocyte cross-talk. J leukocyte biol.

[4] Gerdes N, Zhu L, Ersoy M, Hermansson A, Hjemdahl P, Hu H, et al (2011). Platelets regulate CD4+ T-cell differentiation via multiple chemokines in humans. Thrombosis and haemostasis.

[5] Gibson PH, Cuthbertson BH, Croal BL, Rae D, El-Shafei H, Gibson G, et al (2010). Usefulness of neutrophil/lymphocyte ratio as predictor of new-onset atrial fibrillation after coronary artery bypass grafting. Am J Cardiol.

[6] Toprak E, Bozkurt M, Çakmak BD, Özçimen EE, Silahlı M, Yumru AE, et al (2017). Platelet-to-lymphocyte ratio: A new inflammatory marker for the diagnosis of preterm premature rupture of membranes. J Turkish German Gynecol Assoc.

[7] Yavuzcan A, Caglar M, Ustun Y, Dilbaz S, Yidiz E, Ozbilgec S, et al (2014). Mean platelet volume, neutrophil-lymphocyte ratio and plateletlymphocyte ratio in severe preeclampsia. Ginekologia polska.

[8] Yücel B, Ustun B (2017). Neutrophil to lymphocyte ratio, platelet to lymphocyte ratio, mean platelet volume, red cell distribution width and plateletcrit in preeclampsia. Pregnancy Hypertension: An Intern J Women’s Cardiovascular Heal.

[9] Toptas M, Asik H, Kalyoncuoglu M, Can E, Can MM (2016). Are Neutrophil/ Lymphocyte Ratio and Platelet/Lymphocyte Ratio Predictors for Severity of Preeclampsia?. J Clin Gynecol Obstet.

[10] Kurtoglu E, Kokcu A, Celik H, Sari S, Tosun M (2015). Platelet indices may be useful in discrimination of benign and malign endometrial lesions, and early and advanced stage endometrial cancer. Asian Pac J Cancer Prev.

[11] Klement AH, Hadi E, Asali A, Shavit T, Wiser A, Haikin E, et al (2018). Neutrophils to lymphocytes ratio and platelets to lymphocytes ratio in pregnancy: A population study. PloS one.

[12] Roos N, von Xylander SR (2016). Why do maternal and newborn deaths continue to occur?. Best Pract Res Clin Obstet Gynaecol.

[13] Cleary-Goldman J, Malone FD, Vidaver J, Ball RH, Nyberg DA, Comstock CH, et al (2005). Impact of maternal age on obstetric outcome. Obs Gynecol.

[14] Bateman BT, Mhyre JM, Hernandez-Diaz S, Huybrechts KF, Fischer MA, Creanga AA, et al (2013). Development of a comorbidity index for use in obstetric patients. Obs Gynecol.

[15] Aminu M, Unkels R, Mdegela M, Utz B, Adaji S, Van Den Broek N (2014). Causes of and factors associated with stillbirth in low-and middleincome countries: a systematic literature review. BJOG: Inter J Obs Gynaecol.

[16] Alexander GR, Kogan M, Bader D, Carlo W, Allen M, Mor J (2003). US birth weight/gestational age-specific neonatal mortality: 1995-1997 rates for whites, Hispanics, and blacks. Pediatrics.

[17] Mathews T, MacDorman MF (2007). Infant mortality statistics from the 2004 period linked birth/infant death data set. National vital statistics reports: from the Centers for Disease Control and Prevention, National Center for Health Statistics. National Vital Statistics System.

[18] Guthrie GJ, Charles KA, Roxburgh CS, Horgan PG, McMillan DC, Clarke SJ (2013). The systemic inflammation-based neutrophil–lymphocyte ratio: experience in patients with cancer. Crit. Rev. Oncol/Hematol.

[19] Turkmen K, Erdur FM, Ozcicek F, Ozcicek A, Akbas EM, Ozbicer A, et al (2013). Platelet-to-lymphocyte ratio better predicts inflammation than neutrophil-to-lymphocyte ratio in end-stage renal disease patients. Hemodial Inter.

[20] Gary T, Pichler M, Belaj K, Hafner F, Gerger A, Froehlich H, et al (2013). Platelet-to-lymphocyte ratio: a novel marker for critical limb ischemia in peripheral arterial occlusive disease patients. PLoS One.

[21] Cummings M, Merone L, Keeble C, Burland L, Grzelinski M, Sutton K, et al (2015). Preoperative neutrophil: lymphocyte and platelet: lymphocyte ratios predict endometrial cancer survival. Br J Cancer.

[22] Akgun N, Namli Kalem M, Yuce E, Kalem Z, Aktas H (2017). Correlations of maternal neutrophil to lymphocyte ratio (NLR) and platelet to lymphocyte ratio (PLR) with birth weight. J Mat Fetal Neon Med.

